# Prevalence of Metabolic Syndrome Components in an Urban Mexican Sample: Comparison between Two Classifications

**DOI:** 10.1155/2012/202540

**Published:** 2011-12-06

**Authors:** Irma Isordia-Salas, David Santiago-Germán, Helem Rodrìguez-Navarro, Martín Almaráz-Delgado, Alfredo Leaños-Miranda, Francisco Anaya-Gómez, Gabriela Borrayo-Sánchez, Abraham Majluf-Cruz

**Affiliations:** ^1^Unidad de Investigación Médica en Trombosis, Hemostasia y Aterogénesis, HGR No. 1 “Dr. Carlos Mac Gregor Sánchez Navarro” Instituto Mexicano del Seguro Social, CP 06720, México, DF, Mexico; ^2^Escuela Nacional de Ciencias Biológicas, Instituto Politécnico Nacional, CP 07738, México, DF, Mexico; ^3^Jefe del Laboratorio Clínico del, HGR No. 1 “Dr. Carlos Mac Gregor Sánchez Navarro” Instituto Mexicano del Seguro Social, CP 06720, México, DF, Mexico; ^4^Unidad de Investigación Médica en Medicina Reproductiva, UMAE HGO 4, Instituto Mexicano del Seguro Social, México, DF, Mexico; ^5^Servicio de Medicina Interna, HGR No. 1 “Dr. Carlos Mac Gregor Sánchez Navarro” Instituto Mexicano del Seguro Social, CP 06720, México, DF, Mexico; ^6^Hospital de Cardiología, Centro Médico Nacional Siglo XXI, Instituto Mexicano del Seguro Social, CP 06720, México, DF, Mexico

## Abstract

*Background*. The aim of this study was to examine the prevalence of metabolic syndrome (MS) components in an urban Mexican sample. *Methods*. A total of 854 subjects were included. Anthropometric, blood pressure measurements, clinical data, and overnight fasting blood samples were obtained from all subjects. *Results*. In accordance with definitions by the American Heart Association/ National Heart, Lung, and Blood Institute (AHA/NHLBI) and the International Diabetes Federation (IDF), the prevalence of MS among participants was 59.7 and 68.7%, respectively. The prevalence of MS was higher in women and in individuals older than 45 years of age. More than 40% of the subjects fulfilled four criterions of MS according to both definitions. *Conclusions*. There was a high prevalence of MS components in an urban Mexican sample. Therefore, strong strategies had to be developed for early detection of MS and its components to prevent DMT2 and atherothrombotic complications in these patients.

## 1. Introduction

The original description of the metabolic syndrome (MS) by Reaven [1] consisted of abdominal obesity, insulin resistance, high blood pressure, impaired glucose tolerance or diabetes, hyperinsulinemia and dyslipidemia characterized by elevated triglyceride, and low HDL concentration. All of the features described above are risk factors for atherosclerosis, and thus MS constituted a significant risk for atherothrombosis disease. The World Health Organization in 1998 [2] and the Adult Treatment Panel III (ATP III) in 2001 standardized the definitions of MS [3]. In 2005, The International Diabetes Federation (IDF) formulated a new definition of the MS in a global consensus statement [4]. The prevalence of MS in adults varies from one population to another worldwide. Several studies indicate that, in USA, one-third of adults [5] with an alarming proportion of adolescents and children [6, 7] have the MS. Also a high prevalence of MS in Europe has been demonstrated [8]. It was reported that nondiabetic subjects under 40 years of age had an MS prevalence of 14–41%, depending on the age range [8]. In Mexico, several studies had documented a high prevalence of MS [9, 10], with an increased tendency due to changes in lifestyle behavior (overweight and obesity, physical inactivity, high carbohydrate diets, alcohol, and tobacco consumption) and genetic predisposition. The prevalence of the components of MS is increased in obesity [11] and is associated with atherothrombotic complications in micro- and macrovascular territories [12, 13]. The syndrome is also strongly associated with the increased risk of coronary heart disease and type 2 diabetes mellitus (T2DM) [14–17]. The abnormal metabolic state that accompanies diabetes causes arterial dysfunction. Relevant abnormalities include chronic hyperglycemia, dyslipidemia, and insulin resistance [18, 19]. Atherothrombotic disease is the leading cause of dead worldwide and is the result of genetic and environmental factors [20, 21]. 

We have previously identified genetic variants associated with myocardial infarction and stroke in Mexican young population, in whom components of MS are present [22, 23]. Therefore, the aim of this study was to examine the prevalence of components of syndrome (MS), among Mexican adult population, and to evaluate the genetic participation in this group of patients with high vulnerability to T2DM and atherothrombotic disease.

## 2. Materials and Methods

We conducted a study to identify the prevalence of MS components using two definitions: the IDF and AHA/NHLBI in an urban Mexican sample from Mexico City. Individuals >20 years of age were invited to participate in the study if they were interested to know the risk to develop cardiovascular disease or T2DM. The recruitment period lasted for 1 year (1 May–30 May 2011). All included subjects provided the informed written consent to participate in the study. 

### 2.1. Methods

Subjects were interviewed privately by a physician using pretest questionnaires. The following, demographic and clinical data were collected at the time of the interview: sex, age, cigarette smoking previous diseases, familial history of diabetes, and atherothrombotic disease. 

Waist circumference (WC) was measured at the midpoint between the last rib and the iliac crest with participants standing and wearing only undergarments. Body weight was measured by precision scale while subjects were minimally clothed without shoes. Height was measured in a standing position without shoes using tape meter while the shoulders were in a normal state. Body mass index (BMI) was calculated as weight in kilograms divided by height in meters squared. Patients were defined as overweight with BMI of 25.0–29.9 kg/m^2^, and obese with a BMI ≥30 kg/m^2^. All measurements were taken by the same person. Blood pressure (at rest) was measured with the participant seated. Two readings were taken in 5-minute interval between these two separated measurements, and thereafter the mean of the two measurements was considered to be the participant's blood pressure. The subjects were considered smokers if they were currently smoking (regularly or occasionally, including also former smokers defined as people who stopped smoking at least one year before the examination). A familial history of atherothrombotic disease was defined as acute myocardial infarction (AMI), stroke, or sudden death in a first-degree male relative younger than 55 years of age or a female relative younger than 65 years of age. 

### 2.2. Laboratory Methods

In the morning, after an overnight fast, venous blood was sampled for the measurements of the serum glucose, total and HDL cholesterol, triglycerides, hs-CRP, uric acid, and plasma concentration of fibrinogen. Plasma low-density lipoprotein (LDL) cholesterol was calculated with the equation of Friedewald et al. [24], except when triglycerides exceeded 400 mg/dL. Buffy coat was collected and frozen for genetic studies.

Plasma levels of LDL, fibrinogen, uric acid, hs-CRP, and HbA1c were considered high if they were above 160 mg/dL, 400 mg/dL, 6 mg/dL in women and 7 mg/dL in men, 3.0 mg/L, and 6.5%, respectively.

The study protocol was reviewed and approved by the Human Ethical Committee and Medical Research Council of Instituto Mexicano del Seguro Social and conforms to the ethical guidelines of the 1975 Declaration of Helsinki. Informed written consent was obtained from all subjects before enrollment.

### 2.3. Diagnostic Criteria of the Metabolic Syndrome

The MS was defined according to each of the IDF and AHA/NHLBI definitions as described in Table 1 [4, 25].

### 2.4. Statistical Analysis

Continuous data are expressed as the mean ± standard deviation (SD); categorical data are expressed with percentages. The significance of differences between continuous variables was determined by Student's *t*-test. Differences between categorical variables were determined with the chi-square test. A *P* value <0.05 was considered as statistically significant. All statistical analyses were performed using SPSS (statistical package for the social sciences) statistical software package (version 16: SPSS Inc, Chicago, Il, USA).

## 3. Results

**Figure 1:**
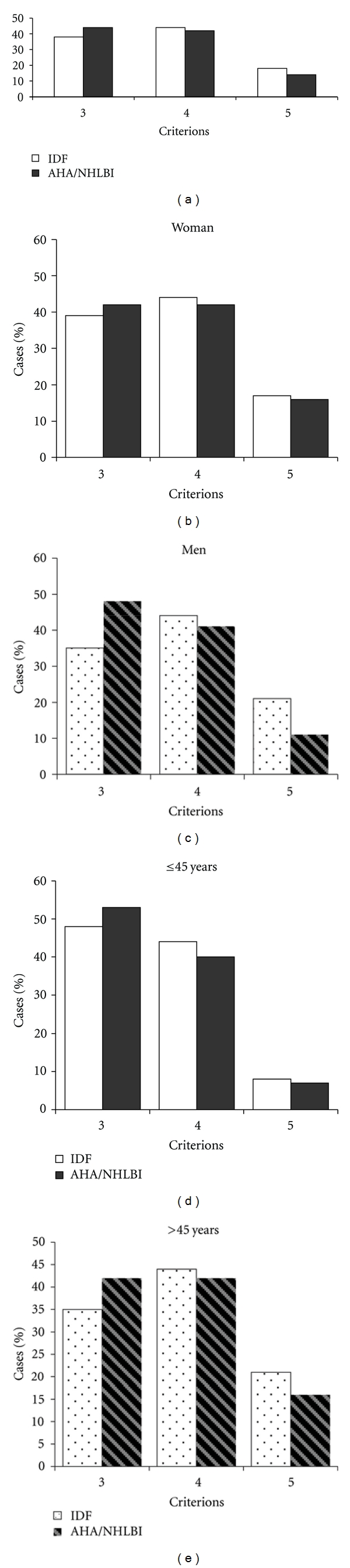
Percent of patients with number of criterions based on IDF y AHA/NHBLI definitions stratified by sex and age. Regardless of the severity of the metabolic syndrome, we found that 43.7% of the individuals fulfilled four criterions when MS was diagnosed by IDF, and 45.3% of the subjects had three components when was used the AHA/NHLBI definition. Similar results were found when we did the analysis stratified by gender. As we expect, in the analysis stratified by age-based MS by IDF and AHA/NHLBI definitions, we found that individuals ≤45 years old fulfilled 3 criterions, whereas four components were present in the older group (43.8%).

A total of 854 subjects were included in this study (see Figure 1). To ascertain whether different definitions may yield different prevalence, MS was diagnosed on the bases of American Heart Association/National Heart, Lung, and Blood Institute (AHA/NHLBI) and International Diabetes Federation (IDF) criterions (Table 1). 

Table 2 shows demographic, clinical, and biochemical features of subjects with MS (MS+), and without MS (MS−). The metabolic syndrome was identified in 607 individuals (407/woman versus 200/men). The mean of age of the subjects MS+ and MS− was similar (53.4 ± 11.0 versus 49.3 ± 13.4, *P* = 0.43), respectively. The body mass index (BMI) was MS+ 29.9 ± 4.8 versus MS− 26.7 ± 4.2, *P* < 0.001. There was a statistical significance in terms of waist circumference (MS+ 97.3 ± 10.8 cm versus MS− 88.5 ± 11.5 cm) (*P* = 0.001). There was a higher triglycerides levels in the group of MS+ compared with MS− (233.8 ± 220.7 versus 129.0 ± 7.3) (*P* < 0.001). The high-density lipoprotein cholesterol (HDL-C) was lower in the group of patients with MS (*P* < 0.001). There was no differences in total cholesterol between both groups (*P* = 0.58). The concentration of fibrinogen, uric acid, and HbA1c was higher in the group of MS+, compared with MS−. 

As it was expected there was a higher frequency of cardiovascular risk factors such as smoking, hypertension, and familial history of atherothrombotic disease in the group of individuals with MS. The prevalence of MS diagnosed based on IDF, AHA/NHLBI, or combination of both definitions was (68.7%, 59.7%, and 57.2%), respectively. 

Table 3 shows the analysis of metabolic syndrome according to the IDF definitions. There was no difference in mean of age between men (53.1 ± 9.5 years) and women (53.7 ± 11.3 years). It was more frequent in women (67.7%) than in men (32.3%). Reduced HDL cholesterol was more frequent in woman (93.2%) compared with the group of men (90.0%). The prevalence of elevated triglycerides was similar in both groups. There was a similar percent of individuals with elevated fasting glucose ≥100 mg/dL or previous diagnosis of T2DM women (64.8%) versus men (67.3%). In the group of women (64.8%) with the criteria before mentioned, 35.8% had glucose level between ≥100 mg/dL and <126/mg/dL, and 29.0% had been already diagnosed with T2DM whereas, in the group of men, 33.1% had ≥100 mg/dL and <126/mg/dL, and 34.2% had been diagnosed with the disease. Hypertension was more common in men (54.7%) than in woman (46.1%). 

Table 4 shows the MS diagnosed based on AHA/NHLBI criterions, and low HDL cholesterol was the most common with a similar frequency in both sexes (men 95.5%) versus (women 93.5%), followed by increased triglyceride levels with higher percentage in men (80.0%) and women (76.0%). There was a slightly high percent of individuals with elevated fasting glucose ≥100 mg/dL or previous diagnosis of T2DM in the group of men (73.5%) versus women (70.4%). Hypertension was the least frequent component with 65.2% in the group of man versus 50.2% in woman. There was a very significant difference in waist circumference between both groups: men (47.0%) versus woman (83%). 

Table 5 shows the MS diagnosed based on IDF definition and stratified by age (≤ y >45 years old). There was a higher percent of men in the group of younger individuals, whereas the women were the predominant gender in the older group. Followed by the waist circumference, the reduced HDL-C was the second most frequent criteria with 94.4% in individuals ≤45 years old versus >45 years (91.5%). We found a significant difference in elevated triglycerides levels between both groups of age, with higher levels in the young group (83.3%) versus the oldest one (71.6%). In terms of individuals with previous diagnosis of T2DM or elevated fasting glucose ≥100 mg/dL, there was a higher percent in the group of older individuals (68.3%) compared to the youngest one (55.6%). The percent of elevated blood pressure was higher in the old group against the youngest one (54.9% versus 27.0%).

Table 6 shows the results of the analysis of individuals with MS diagnosed based on the AHA/NHLBI stratified by age. The female group was predominant in both groups of age in the group of younger women (61.9%) and (69.4%) in the oldest one. The most common criterion was reduced HDL-C with 97.0% in the youngest group versus 93.4% in the old one. In contrast, as we expected we found elevated triglycerides levels in both groups, with higher percent in the group ≤45 years of age (86.1%) compared with the oldest group (75.0%). There was a slight difference in the percent of waist circumference between the groups (≤45 years, 74.3% versus >45 years, 71.6). Increased fasting glucose ≥100 mg/dL or previous diagnosis of T2DM was found in 67.3% of young individuals versus 72.4% <45 years of age. As it was expected, there was a higher percent of individuals with high blood pressure or previous diagnosis of hypertension in the group of >45 years of age (61.1%) versus (28.7%) in the youngest group.

We want to point out that we identified an increased level of fasting overnight glucose ≥100 mg/dL and <126 mg/dL in the group of individuals ≤45 years of age when MS was diagnosed by IDF criterions (38.9%), versus >45 years of age (33.8%), and when diagnosed by AHA/NHLBI ≤45 years (46.5%) versus >45 years old (35.5%) (data not shown).

## 4. Discussion

Patients with MS die from complication of T2DM and atherothrombotic disease such as acute myocardial infarction and stroke. In previous studies, a high frequency of metabolic syndrome (MS) has been identified in our population. Therefore, the aim of this study was to identify phenotypic, specific and genotypic profile that may help to improve the prevention of T2DM, dyslipidemia, and atherothrombotic complication such as AMI and Stroke. Although the final results with respect to this principal aim are still awaited, the study clearly confirms that an early screening should be performed in individuals anticipated to be at risk of T2DM, dyslipidemia, and obesity by their primary care physician and should be treated in metabolic syndrome clinic.

The MS was present in 68.7% of the total sample, according to IDF definitions versus 59.7%, compared with AHA/NHLBI. In 57.2% of the individuals the MS was diagnosed base by either one definition IDF or AHA/NHLBI. We identify more individuals with MS by IDF classification compared to AHA/NHLBI. This result is probable due to the waist circumference lower cutoff applied by the IDF. In contrast, by AHA/NHLBI, we found an increased percentage of individuals with hypertension, dyslipidemia, and high levels of glucose, compare to IDF classification, because the waist circumference is not an absolute required criterion. This combination of criterions might represent a different severity of the MS between patients classified by one or another.

Those results are in agreement with those previously reported by Rojas et al. [26], who identified a less frequency of the syndrome by AHA/NHLBI compared with IDF in our population, but are in disagreement with those obtained by other investigators in American population >20 years [4]. 

Another relevant issue found in this study is the high prevalence of the numbers of criterions in each subject from this sample stratified by groups of age and gender by both classifications IDF and AHA/NHLBI. In the present study, we found a high percent of individuals who fulfilled four or more criterions. However, for the IDF classification, the waist circumference was the most frequent criterion, followed by lowered HDL-C and increased level of triglycerides, whereas for the AHA/NHLBI, the more frequent component was HDL-C, triglycerides, and waist circumference. The most important is that all three criterions are associated with an increased risk for cardiovascular disease, and they are frequently present in the same individual.

In approximately 42.2% of the individuals ≤45 years of age, who were diagnosed with MS by either classification, IDF, or AHA/NHLBI, registered glucose levels were between ≥100 mg/dL and 125 mg/dL. The most important thing is that they were not under lowering glucose therapy, because they did not have a recent blood glucose test. 

On the other hand, as we expect there was a higher percent of individuals with T2DM >45 years old (35.7%) compared with the subjects ≤45 years old (18.7%) in both groups of patients diagnosed with either definition IDF or AHA/NHLBI. Those results corroborated that insulin resistance (IR) is more frequent in young individuals, whereas T2DM is predominant in older individuals. Therefore, an early detection and lifestyle changes have to be implemented in young individuals to avoid the development of chronic disease such as T2DM and atherothrombotic disease complications such acute myocardial infarction and stroke.

Also, similar results were obtained when we analyzed the triglycerides levels by age.

In 73.97% of individuals with MS diagnosed by either definitions, IDF or AHA/NHLBI were found to have an increased triglycerides levels (≥150 mg/dL), and HDL-C below normal ranges was diagnosed. Most of them had history of dyslipidemia, but only 19.6% were under lowering lipid therapy. In this particular case is necessary a rigorous and constant monitoring in patients with previous history of dyslipidemia. 

Several new features have been added to the syndrome over time. These include elevated plasminogen activator inhibitor-1 (PAI-1) and C-reactive protein (CRP) concentrations. These features were added on the basis that they were frequently found in association with the metabolic syndrome. These features are probably related to both insulin resistance and obesity [27]. In the present study, we failed to identify, an association between high levels of hs-CRP and MS, and those results are in agreement with those previously published by Han et al. [28]. 

In contrast, we have previously reported that higher levels of plasma PAI-1 represent a risk factor for development of ST elevation acute myocardial infarction (STEAMI) [29]. 

Therefore, the following step will be determinate PAI-1 levels in the same group of patients with MS. Those results probabley allow us to include a new atherothrombotic marker in this type of patients in our population.

A previous study by Madrid-Miller [30] explores the clinical impact of MS in patients with acute coronary syndrome (ACS). The MS was more frequent in older patients with ACS and was associated with poorer in-hospital outcomes. 

Since most patients with T2DM die from complications of atherosclerosis, they should receive intense preventive interventions proven to reduce their cardiovascular risk.

Using the AHA/NHLBI definitions, MS is present in 82.3 of self-reported coronary heart diseases (CHD), 87.5% of type 2 diabetes cases, 43.1% self-reported with high triglycerides, 47.6% of subjects with low HDL-cholesterol levels, and 70.5% of adults with hypertension [26]. 

That percentage provides a gross estimate of the contribution of MS to the outcomes mentioned above and justifies the screening of MS components in persons with those conditions. These results will be useful for updating local guidelines for the prevention and treatment of specific chronic disorders, which requires a multidisciplinary team approach that implements lifestyle changes and a combination of drugs (when appropriate). The primary goal of clinical management in individuals with MS is to reduce the risk of clinical atherosclerosis disease.

Therefore, our data describes the significant challenges that MS represents to our health system. The Mexican health system should develop specific management programs for all identified cases; failure to identify or treat cases of MS will result in a considerable increase in new forms of T2DM and/or CHD.

## 5. Conclusions

Our results identify that more than 40% of individuals with MS has four or more criterions, which represent the severity of the syndrome among urban Mexican sample. Therefore, strong strategies had to be developed for early detection of MS and its components in order to prevent DMT2 and atherothrombotic complications in these patients with myocardial infarction and stoke.

## Figures and Tables

**Table 1:** Criteria for Clinical diagnosis of the metabolic syndrome by the IDF and AHA/NHLBI definitions.

The IDF definition	The AHA/NHLBI definition
Central obesity: defined as waist circumference	
≥90 cm for Asian men and ≥80 cm for Asian woman	
plus any two of the following four criteria	Three or more of the following

	Central obesity: defined as waist circumference
	≥102 cm for men and ≥88 for woman

Raised fasting plasma glucose (FPG): ≥100 mg/dL,	Raised fasting plasma glucose (FPG): ≥100 mg/dL
or previously diagnosed type 2 diabetes	or previously diagnosed type 2 diabetes

Raised triglycerides ≥150 mg/dL,	Raised triglycerides ≥150 mg/dL
or specific treatment for this lipid abnormality	or specific treatment for this lipid abnormality

Reduced HDL cholesterol:	Reduced HDL cholesterol:
<40 mg/dL in males and <50 mg/dL in females	<40 mg/dL in males and <50 mg/dL in females

Elevated blood pressure ≥130/≥85 mm Hg, or	Elevated blood pressure ≥130/≥85 mm Hg, or
previous medical diagnosis of hypertension	previous medical diagnosis of hypertension

**Table 2:** Demographic, clinical, and biochemical features of subjects with and without metabolic syndrome included in the study.

	MS+	MS−
Subjects *n* (%)	607 (70%)	247 (30%)
Gender *n* (%)		
Man	200 (33.0%)	74 (30.0%)
Women	407 (67.0%)	173 (70.0%)
Age (years)	53.4 ± 11.0	49.3 ± 13.4
Body mass index (kg/m^2^)	29.9 ± 4.8	26.7 ± 4.2
Waist circumference (cm)	97.3 ± 10.8	88.5 ± 11.5
Triglycerides (mg/dL)	233.8 ± 220.7	129.0 ± 7.3
HDL-C (mg/dL)	36.02 ± 9.0	48.0 ± 13.7
Total cholesterol (mg/dL)	211.8 ± 61.5	205.8 ± 42.4
FPG (mg/dL)	158.3 ± 48.1	94.8 ± 16.9
Fibrinogen (mg/dL)	377.0 ± 89.2	3 69.1 ± 71.5
Uric Acid (mg/dL)	5.4 ± 2.8	3.9 ± 1.3
hs-CRP (mg/dL)	1.02 ± 0.5	0.78 ± 0.22
HbA1c (%)	5.0 ± 2.6	4.3 ± 2.1
Currents smoking *n* (%)	136 (22.4)	45 (18.2)

Blood pressure ≥130/≥85 mmHg or previous diagnosis of hypertension		

*n* (%)	301 (49.58)	
Previously diagnosed hypertension *n* = (%)	282 (46.45)	23 (30.2)
New cases of hypertension *n* = (%)	19 (3.13)	

Elevated fasting glucose ≥100 mg/dL or previous diagnosis of T2DM		

*n* (%)	398 (65.56)	
Previously T2DM *n* = (%)	165 (27.18)	33 (30.2)
New cases of T2DM *n* = (%)	26 (4.28)	

No T2MD patients with FPG ≥100 mg/dL–125 mg/dL *n* = (%) 209 (34.43)		

FH of AT *n* (%)	112 (18.45)	30 (14.5)

HDL-C = high-density lipoprotein cholesterol, FG = fasting glucose, hsCRP = high sensitivity C protein reactive, HbA1c = glycosylate hemoglobin, T2DM = type 2 diabetes mellitus, IDF = International Diabetes Federation, AHA/NHLBI = American Heart Association/National Heart, Lung, and Blood Institute, FH of EAT = familial history of atherothrombosis.

**Table 3:** Prevalence (%) of MS and its components to IDF definition, by gender.

Measure (any 3 of 5 constitute diagnosis of MS)	Men	Women
	Total = 587	
	*n* = 190 (32.3%)	*n* = 397 (67.7%)
Age (years)	53.1±	53.7±
Waist circumference *n* (%)	190 (100%)	397 (100%)
Men ≥90 cm		
Women ≥80 cm		
Reduced HDL-C *n* (%)	171 (90.0%)	370 (93.2%)
Men <40 mg/dL		
Women <50 mg/dL		
Triglycerides ≥150 mg/dL or medical treatment of elevated TG *n* (%)	142 (74.7%)	293 (73.8%)
Elevated fasting glucose ≥100 mg/dL or previous diagnosis of T2DM	128 (67.3 %)	257 (64.8%)
Blood pressure ≥130/≥85 mmHg or previous diagnosis of hypertension *n* (%)	104 (54.7%)	183 (46.1%)

**Table 4:** Prevalence (%) of MS and its components to AHA/NHLBI definition, by gender.

Measure (any 3 of 5 constitute diagnosis of MS)	Men	Women
	Total = 510	
	*n* = 155 (30.4)	*n* = 355 (69.6%)
Age (years)	53.6±	53.9±
Reduced HDL-C *n* (%)	148 (95.5%)	332 (93.5%)
Men <40 mg/dL		
Women <50 mg/dL		
Triglycerides ≥150 mg/dL or medical treatment of elevated TG *n* (%)	124 (80.0%)	270 (76.0%)
Elevated fasting glucose ≥100 mg/dL or previous diagnosis of T2DM	114 (73.5%)	250 (70.4%)
Blood pressure ≥130/≥85 mmHg or previous diagnosis of hypertension *n* (%)	101 (65.2%)	178 (50.2%)
Waist circumference *n* (%)	73 (47.0%)	295 (83.0%)
Men ≥102 cm		
Women ≥88 cm		

**Table 5:** Prevalence (%) of MS and its components to IDF definition, by age.

Age (years)	≤45	>45
	Total = 587	
	*n* = 126	*n* = 461
Sex (%)		
Men	38.1	30.6
Women	61.9	69.4
Waist circumference *n* (%)	126 (100)	461(100)
Men ≥90 cm, women ≥80 cm		
Reduced HDL-C *n* (%)	119 (94.4)	422 (91.5)
Men <40 mg/dL		
Women <50 mg/dL		
Triglycerides ≥150 mg/dL or medical treatment of elevated TG *n* (%)	105 (83.3)	330 (71.6)
Elevated fasting glucose ≥100 mg/dL or previous diagnosis of T2DM	70 (55.6)	315 (68.3)
Blood pressure ≥130/≥85 mmHg or previous diagnosis of hypertension *n* (%)	34 (27.0)	253 (54.9)

**Table 6:** Prevalence (%) of MS and its components to AHA/NHLBI definition, by age.

Age (years)	≤45	>45
	Total = 510	
	*n* = 101	*n* = 409
Sex	53.1±	53.7±
Men	38.1	30.6
Women	61.9	69.4
Waist circumference *n* (%)	75 (74.3)	293 (71.6)
Men ≥102 cm, women ≥88 cm		
Reduced HDL-C *n* (%)	98 (97.0)	382 (93.4)
Men <40 mg/dL		
Women <50 mg/dL		
Triglycerides ≥150 mg/dL or medical treatment of elevated TG *n* (%)	87 (86.1)	307 (75.0)
Blood pressure ≥130/≥85 mmHg or previous diagnosis of hypertension *n* (%)	29 (28.7)	250 (61.1)
Elevated fasting glucose ≥100 mg/dL or previous diagnosis of T2DM	68 (67.3)	296 (72.4)
